# Case Report: Laparoscopy-assisted resection for intra-abdominal gossypiboma masquerading as a jejunal tumor (with video)

**DOI:** 10.3389/fonc.2023.1326032

**Published:** 2023-11-28

**Authors:** Yihui Han, Wenming Yang, Wenshu Dai, Qin Ma, Tao Yuan, Yun Yang, Yanrong Lu, Bo Zhang, Mingming Zhang

**Affiliations:** ^1^Department of General Surgery, West China Hospital, Sichuan University, Chengdu, China; ^2^Gastric Cancer Center, West China Hospital, Sichuan University, Chengdu, China; ^3^Division of Gastrointestinal Surgery, Department of General Surgery, West China Hospital, Sichuan University, Chengdu, China; ^4^Key Laboratory of Transplant Engineering and Immunology, National Health and Family Planning Commission (NHFPC), West China Hospital, Sichuan University, Chengdu, China; ^5^Department of Anesthesiology, West China Hospital, Sichuan University, Chengdu, China; ^6^Colorectal Cancer Center, West China Hospital, Sichuan University, Chengdu, China

**Keywords:** intra-abdominal gossypiboma, tumor, iatrogenic complication, differential diagnosis, laparoscopic surgery

## Abstract

**Introduction:**

Intra-abdominal gossypiboma, a cotton-based retained foreign body after an abdominal surgery, is associated with various clinical manifestations and complications. Its infrequent occurrence and unpredictability make its early diagnosis particularly challenging. We herein present an atypical case of intra-abdominal gossypiboma mistaken for a jejunal tumor.

**Case presentation:**

A 33-year-old female presented to the emergency room with an acute episode of progressive abdominal pain and distention, nausea, and vomiting for 20 hours. She had undergone an urgent cesarean section due to fetal tachycardia seven years prior. The initial diagnosis of small bowel obstruction (SBO) due to a jejunal tumor was established by computed tomography. Subsequent to successful medical management of the SBO, a laparoscopy-assisted resection of the mass and the adherent jejunal segment was conducted, culminating in a primary side-to-side jejunojejunostomy. Examination of the excised tissue revealed an approximately spherical fibrous mass, 6 × 6 × 5 cm in dimension, embedded in the jejunal wall, housing a 20 × 20-cm gauze. Postoperative recovery and routine follow-up ensued without complications.

**Conclusion:**

In light of this case, the need for clinicians to maintain an elevated awareness and suspicion of gossypiboma should be accentuated when evaluating an intra-abdominal mass, especially in patients with a prior history of high-risk laparotomy. Laparoscopic surgery stands out as a technically proficient and minimally invasive strategy for diagnosing and treating intra-abdominal gossypiboma. Besides, it is imperative to emphasize the importance of meticulous surgical procedures and postoperative protocols to prevent such oversights, reaffirming the need for consistent intraoperative counts and checks of surgical items.

## Introduction

Gossypiboma, derived from “gossypium” (Latin for cotton) and “boma” (Swahili for place of concealment), was first documented by Wilson in 1884 (1). It represents a rare yet entirely preventable iatrogenic complication (2). By definition, a gossypiboma is a cotton-based mass within a body cavity that results from the body’s reaction to a sponge or gauze inadvertently retained post-surgery. In the United States, the incidence varied from 1 in 1,000 – 1,500 to 1 in 8,801 - 18,760 inpatient operations in the past decades (3). Due to malpractice claims and medicolegal consequences involved, the actual incidence of gossypiboma remains unknown and definitely underestimated (4). The gossypiboma formation are reported to be significantly associated with emergency surgery, unplanned changes in procedure, morbid obesity, and multiple major procedures done in a single operation or cases in which multiple surgical teams were involved (3, 5, 6).

The abdominopelvic cavity was the most frequent body cavity where the foreign body left (54%), followed by vagina (22%), thorax (7%), and others (17%; including the spinal canal, face, brain, and extremities) (3). Notably, while gossypibomas are less likely to occur in laparoscopic or robotic procedures, their prevalence is higher in laparotomy (7). Intra-abdominal gossypiboma patients can present to hospital with various clinical manifestations and even significant morbidity, such as ileus, perforation, abscess collection, fistula formation, and sepsis (8, 9). Due to rare and unanticipated, precise preoperative diagnosis of intra-abdominal gossypiboma is extremely challenging (10).

In alignment with the principles of the CAse REport (CARE) guidelines (11), we herein describe an unusual case of intra-abdominal gossypiboma complicated by ileus and masquerading as a jejunal tumor, which was treated successfully by laparoscopy-assisted resection. In light of this case, it is imperative to emphasize the importance of meticulous surgical procedures and postoperative protocols to prevent such oversights, reaffirming the need for consistent intraoperative counts and checks of surgical items.

## Case presentation

In November 2022, a 33-year-old Han Chinese female patient was referred to our emergency room with progressive abdominal pain (colicky in nature; sudden onset, continuous, crushing in character) and distention, nausea, and vomiting for 20 hours. She had undergone an urgent cesarean section because of fetal tachycardia 7 years previously. There were no underlying comorbidities or family history of malignancies with her. On admission, she was subfebrile with slightly abnormal vital signs (Temperature, 37.5°C; Blood pressure, 108/72 mmHg; Heart rate, 102 beats per minute; Respiratory rate, 19 beats per minute). Physical examination showed abdominal distention, slight rebound tenderness in the periumbilical area, and a painless palpable mass in the lower quadrant with metallic bowel sounds. The result of digital rectal examination was unremarkable.

**Figure 1:**
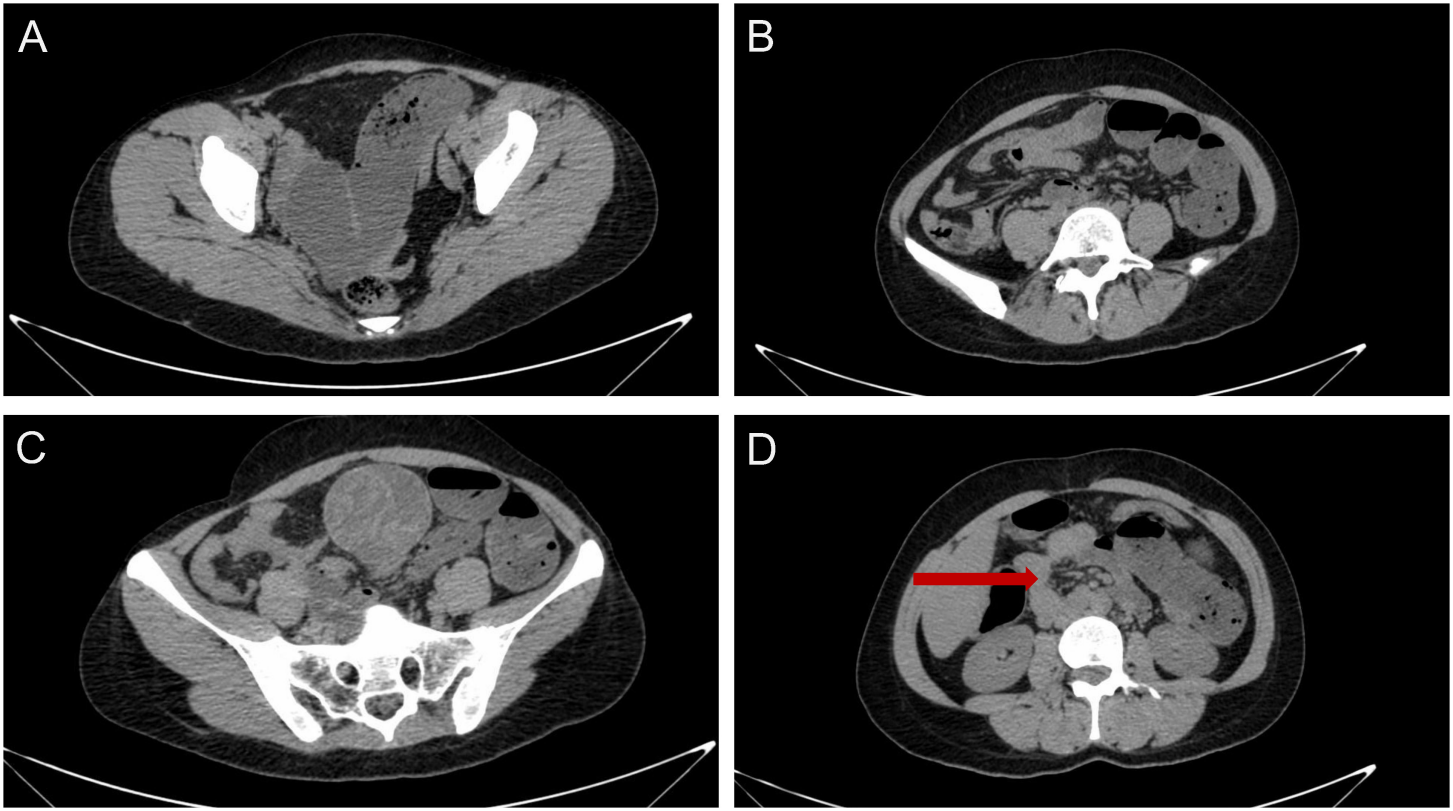
Computed tomography of the abdomen and pelvis indicated **(A, B)** dilated proximal bowel loops and multiple air-fluid levels due to **(C)** a 6.1 × 5.7-cm, approximately spherical, well-delineated heterogeneous cystic and hyperdense jejunal mass and **(D)** mild intestinal volvulus, without indicators of intestinal ischemia or necrosis.

Blood tests indicated a systemic infection with an elevated total leukocyte count (13.2 ×10^9/L; reference range, 3.5 – 9.5 × 10^9/L), percentage of neutrophils (91%; reference range, 40 – 75%), and C-reaction protein (36 mg/L; reference range, <5 mg/L). Contrast-enhanced computed tomography (CT) of the abdomen and pelvis demonstrated dilated proximal bowel loops and multiple air-fluid levels due to a 6.1 × 5.7-cm, approximately spherical, well-delineated heterogeneous cystic and hyperdense jejunal mass and mild intestinal volvulus, without indicators of intestinal ischemia or necrosis (**Figure 1**). There were no metastases to the liver, greater omentum, or peritoneum detectable on radiologic imaging.

The preliminary suspected diagnosis of SBO due to a jejunal tumor and mild intestinal volvulus was established by above clinical findings. The physical condition of the patient was optimized and elective laparoscopic exploration was then scheduled for her (12). Intraoperatively, a solitary jejunal mass was found to be the possible lead point of intestinal volvulus and subsequent SBO. Therefore, laparoscopy-assisted resection surgery was performed according to the following steps (13, 14): (1) adhesionlysis and complete mobilization of the intra-abdominal mass; (2) extracorporeal resection with wide surgical margin of the mass and segmental jejunum (15); (3) primary side-to-side jejunojejunostomy; (4) check for a safe anastomosis and no intestinal volvulus under laparoscopy (16); (5) peritoneal irrigation and drainage placement (**Supplementary Material Figure S1**; **Supplementary Material Video S1**). The procedure lasted 115 minutes, with estimated blood loss of 20 ml.

**Figure 2:**
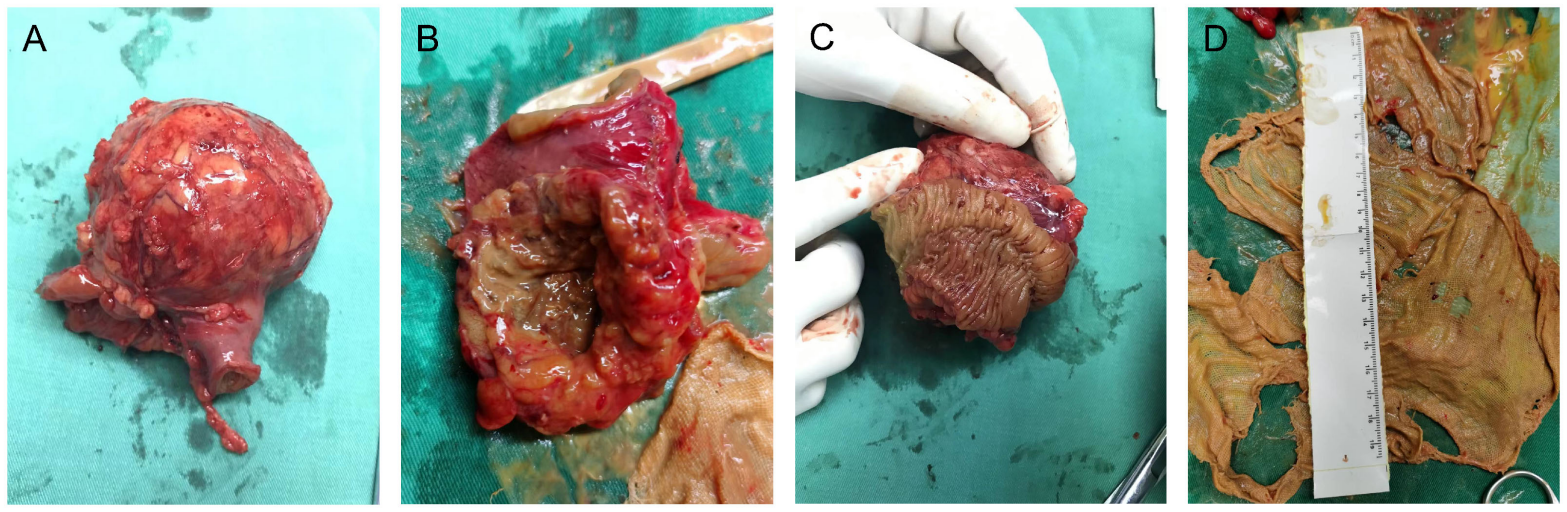
Gross examination of the surgical specimen demonstrated a 6 × 6 × 5-cm, approximately spherical mass of fibrous hyperplasia adherent to the jejunal wall **(A–C)**, which contained a 20 × 20-cm gauze **(D)**.

**Figure 3:**
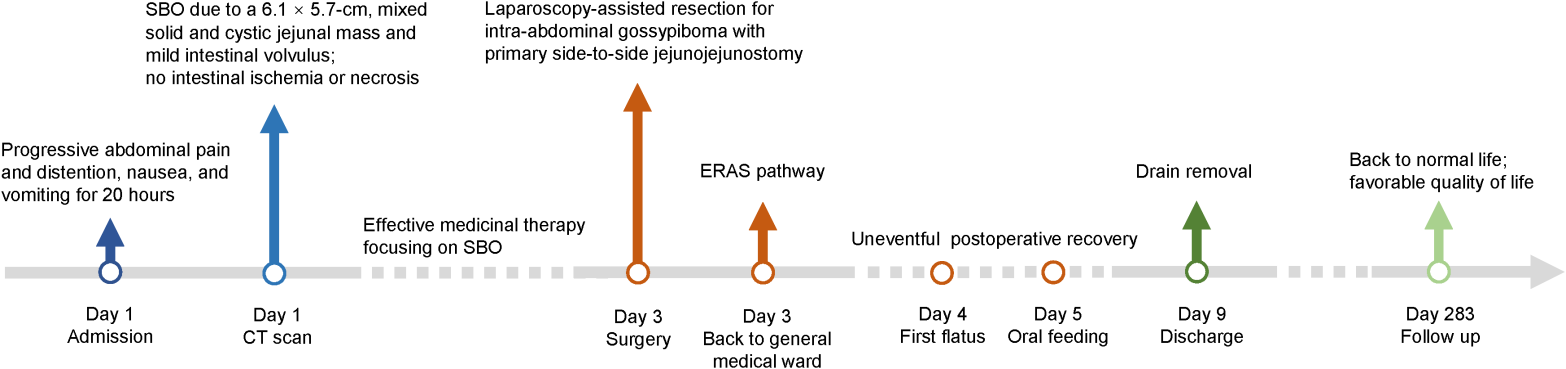
Timeline of the case presentation with relevant data from the episode of care. SBO, small bowel obstruction; CT, computed tomography; ERAS, enhanced recovery after surgery.

Gross examination of the resected specimen demonstrated a 6 × 6 × 5 cm in dimension, approximately spherical mass of fibrous hyperplasia adherent to the jejunal wall, which contained a 20 × 20-cm surgical gauze (**Figure 2**). Given potential medicolegal consequences, histopathologic examination was not conducted. Because of the possibility of negative result under effective antibiotics treatment preoperatively, the gauze was not sent to bacteriology examination. Postoperatively, the patient was transferred back to the general medical ward. Complying with the pathway of enhanced recovery after surgery, the course of postoperative rehabilitation was uneventful, with first flatus on postoperative day (POD) 1 and oral feeding beginning on POD 2. The patient was discharged on POD 6 without any complications. So far, the regular follow-up of 9 months has been unremarkable. The timeline with corresponding clinical data from the period of care is shown in **Figure 3**.

## Discussion

A systematic literature search was conducted in the PubMed database utilizing medical subject headings and text words related to “intra-abdominal gossypiboma” to acquire relevant case reports published till October 13, 2023. The search strategy and syntax for PubMed database are shown in **Supplementary material Table S1**. The studies with initial misdiagnosis as intra-abdominal tumor met the eligible criteria. Systematic reviews, case series with unavailable individual patient data, and articles with non-English languages or animal subjects were excluded. The general characteristics of the enrolled studies were extracted and entered into a preplanned electronic form (**Table 1**). The pooled data were summarized in a narrative and descriptive way.

**Table 1:** General characteristics of the included case reports regarding intra-abdominal gossypiboma with initially suspected diagnosis as tumor.

First author	Year	Country	Age & Sex	Previous operation	Interval	Clinical presentation	Diagnostic method	Initial diagnosis	Treatment
Jason RS (17)	1979	USA	39 M	exploratory laparotomy	12 y	intermittent abdominal pain associated with a firm, non-tender epigastric mass	upper GI series	pancreatic carcinoma	surgical removal of the sponge & cystogastrostomy for the lesser sac cyst
Cheng TC (18)	2007	China	61 M	total gastrectomy	8 y	intermittent right upper abdominal pain	CT	recurrent gastric cancer	surgical resection
Yamamura N (19)	2008	Japan	78 M	distal gastrectomy; cholecystectomy	40/15 y	asymptomatic	CT, EUS, MRI	gastric GIST	surgical resection
Akbulut S (20)	2011	Turkey	51 M	laparotomy	8 y	colicky abdominal pain, intermittent abdominal distention, constipation, nausea, and vomiting	CT	ileal tumor	segmental ileal resection
Bulus H (21)	2011	Turkey	67 F	laparotomy for acute cholecystitis	5 y	recurrent, vague, central abdominal pain	US, CT	small bowel GIST	segmental jejunal resection
Cheon JW (22)	2011	Korea	78 F	partial gastrectomy	30 y	sudden onset epigastric pain	CT, EGD, US	gastric GIST	wedge resection of gastric fundus
Kawamura Y (23)	2012	Japan	41 F	cesarean section	2 y	intermittent abdominal pain	CT, MRI	GIST	resection of the tumor together with adherent small bowel
Shen HP (24)	2012	China	68 F	total hysterectomy	25 y	abdominal fullness, constipation, and a palpable protruding mass from the rectum	colonoscopy, US	adnexal malignancy	surgical resection
George AJP (25)	2014	India	46 F	right nephrectomy	8 y	continuous right flank pain	CT	retroperitoneal tumor	surgical resection
Eken H (26)	2016	Turkey	62 F	myomectomy	20 y	stomachache, distention, and constipation	CT, MRI	mesenchymal malignancy	resection of the bulk together with adherent small bowel
Singla N (27)	2016	USA	75 F	hysterectomy	NA	lower abdominal pain and fullness	CT	urachal tumor	resection of the urachal mass with a cuff of adherent dome of bladder
Nishimura N (28)	2017	Japan	67 F	hysterectomy	24 y	hematochezia	colonoscopy, CT	transverse colon tumor	resection of the mass together with the transverse colon
Zhang H (29)	2017	China	32 F	cesarean section	8 y	intermittent left lower abdominal pain	US, CT	ovarian teratoma	resection of the mass together with adhesive small bowel
Oran E (30)	2018	Turkey	28 F	open cholecystectomy	3 y	abdominal pain	CT, MRI	pancreatic carcinoma	surgical resection
Oran E (30)	2018	Turkey	36 F	cesarean section (twice)	13/15 y	painful mass in the left lower quadrant	US, CT	left ovarian tumor	surgical resection
Arikan Y (31)	2019	Turkey	74 M	open pyelolithotomy	20 y	asymptomatic	MRI	left kidney malignancy	left partial nephrectomy
Boghratian AH (32)	2020	Iran	30 F	mini gastric bypass	2 mo	nausea, non-bloody vomiting, and abdominal pain	CT	small bowel malignancy	endoscopic removal
Celik H (33)	2021	Turkey	36 F	ectopic pregnancy operation	7 y	severe abdominal pain and distension	US, CT	intra-abdominal tumor	resection of the mass together with adherent small bowel
Hajri A (34)	2021	Morocco	65 F	open cholecystectomy	29 y	nausea and vomiting, loss of appetite and weight loss	EGD, CT	GIST	atypical hepatectomy of segment 3
Modrzejewski A (35)	2023	Poland	49 F	myomectomy	2 y	urinary incontinence	CT	sigmoid colon tumor	NA

M, male; F, female; y, years; mo, months; GI, gastrointestinal; CT, computed tomography; EUS, endoscopic ultrasonography; MRI, magnetic resonance imaging; GIST, gastrointestinal stromal tumor; US, ultrasonography; EGD, esophagogastroduodenoscopy; NA, not available.

As a result, 233 articles were screened for further potential. Then, 20 cases with intra-abdominal gossypibomas which were initially misdiagnosed as tumors in 19 literature (between 1979 to 2023) met the inclusion criteria (17–35). Overall, the relevant case reports focusing on this special population emerged rapidly in the past decade. These events of initial misdiagnosis proved to occur mostly in Asian countries (15/19), especially Turkey. Despite the risk of publication bias, relatively insufficient healthcare resources in transitioning economies could definitely contribute to higher incurrence rate of intra-abdominal gossypiboma to some extent. Female patients accounted for 75% of the included cases (15/20). Previous obstetrics and gynecology surgery remained the leading cause of intra-abdominal gossypiboma (9/20), followed by open cholecystectomy (4/20), gastrectomy (4/20), urinary operations (2/20), and other laparotomies. The interval between the previous procedures to the present diagnosis of intra-abdominal gossypiboma ranged from 2 months to 40 years. The most common diagnostic tools were CT and ultrasonography (US). In terms of tumor, the predominant misdiagnosis for these patients as well as our reported case was gastrointestinal stromal tumor (GIST).

The optimal approach to dealing with this iatrogenic surgical complication is prevention. For surgeries associated with high risk of retained surgical sponge (RSS) as mentioned above, repeated sponge counting at the key time point (such as beginning and ending of the operation, handover of surgical team, closure of the peritoneum, and every 3 hours) should be advocated and emphasized (2). All the surgeons, assistants, and operating theater nurses should take a meticulous and responsible attitude towards the patient’s life and postoperative quality of life. On the other hand, the application of radiopaque marker and quick response code within the surgical sponge is highly advisable (36–38). However, routinely postoperative plain films to identify RSS is not recommended. Small sponges should be abandoned during laparotomy while surgical compresses should be employed only intraperitoneally and one by one. A thorough intraperitoneal exploration prior to closure of the peritoneum is also crucial to minimize the risk of RSS. In a word, the prevention of RSS requires a high sense of responsibility, standardized clinical practice, diversified knowledge, and shared information.

As mentioned above, intra-abdominal gossypibomas are associated with unpredictable clinical presentations and the possibility of a long interval between the previous surgery and current episode (9). It may be discovered most frequently by a different surgeon rather than the one who did the previous procedure. It is critical for clinicians to hold a high index of suspicion of intra-abdominal gossypiboma when evaluating the episode of new symptoms in these patients with a distant history of high-risk laparotomy. Radiologic examinations (including CT, US, and MRI) can help establish preliminary diagnosis (10). For intra-abdominal gossypiboma presenting as a mass, the common differential diagnoses include GIST, tumor of the small bowel and colon, retroperitoneal tumor, and intra-abdominal abscess. Noticeably, the gossypiboma and abscess can co-exist in the same patient. The role of percutaneous biopsy under the guidance of US or CT and histopathologic examination in the diagnosis of an intra-abdominal mass should be emphasized, once a malignancy tumor cannot be ruled out (27).

Furthermore, gastrointestinal endoscopy, as well as cystoscopy and ureteroscopy, can be applied to identify transmural migration and sometimes remove the RSS (32, 39). For a long time, redo exploratory laparotomy remains the frequent and vital method to finally confirm the diagnosis and treat this specific patient population. However, with minimal invasion and improved visualization, laparoscopic and robotic-assisted approaches have been placed high hopes in the management of intra-abdominal gossypiboma, especially with preoperatively ambiguous diagnosis (40, 41).

In conclusion, our case report accentuates the need for clinicians to maintain an elevated awareness and suspicion of gossypiboma when evaluating an intra-abdominal mass, especially in patients with a prior history of high-risk laparotomy. Laparoscopic surgery stands out as a technically proficient and minimally invasive strategy for diagnosing and treating intra-abdominal gossypiboma.

## Data availability statement

The original contributions presented in the study are included in the article/**Supplementary Material**. Further inquiries can be directed to the corresponding author.

## Ethics statement

The studies involving humans were approved by Biomedical Ethical Committee of West China Hospital, Sichuan University. The studies were conducted in accordance with the local legislation and institutional requirements. The participants provided their written informed consent to participate in this study. Written informed consent was obtained from the individual(s) for the publication of any potentially identifiable images or data included in this article.

## Author contributions

YH: Writing – original draft, Project administration. WY: Writing – original draft, Project administration. WD: Writing – original draft. QM: Writing – review & editing, Data curation, Investigation, Visualization. TY: Writing – review & editing, Formal Analysis, Software. YY: Writing – review & editing, Formal Analysis, Software. YL: Writing – review & editing, Supervision. BZ: Supervision, Writing – review & editing, Conceptualization, Funding acquisition, Methodology. MZ: Supervision, Writing – review & editing, Conceptualization, Funding acquisition, Methodology.

## Supplementary material

Supplementary Figure 1 The flow diagram of the surgical procedure, extracorporeal resection of the mass and segmental jejunum with primary side-to-side jejunojejunostomy.

Supplementary Video 1 Laparoscopy-assisted resection for intra-abdominal gossypiboma masquerading as a jejunal tumor.
