# Supplementary material for: Case Report: Laparoscopy-assisted resection for intra-abdominal gossypiboma masquerading as a jejunal tumor (with video)
Source: Front Oncol. 2023 Nov 28;13:1326032. doi: 10.3389/fonc.2023.1326032 (PMC10715586; doi:10.3389/fonc.2023.1326032)
Supplement: Supplementary file 1 [file Table_1.docx]

| **Search** | **Search terms** |
| --- | --- |
| #1 | gossypiboma[tiab] OR textiloma[tiab] OR gauzoma[tiab] OR gauzeoma[tiab] OR cottonballoma[tiab] OR cottonoid[tiab] OR muslinoma[tiab] OR retained surgical sponge[tiab] |
| #2 | abdominal[tiab] OR intra-abdominal[tiab] OR intraabdominal[tiab] OR intrabdominal OR peritoneal[tiab] OR intra-peritoneal[tiab] OR intraperitoneal[tiab] |
| #3 | #1 AND #2 |
| #4 | animal[tiab] OR mouse[tiab] OR mice[tiab] OR rat[tiab] OR rabbit[tiab] OR dog[tiab] |
| #5 | #3 NOT #4 |

**TABLE S1.** **Search strategy for PubMed database.**
