# Supplementary figures and images for: Case Report: Laparoscopy-assisted resection for intra-abdominal gossypiboma masquerading as a jejunal tumor (with video)
Source: Front Oncol. 2023 Nov 28;13:1326032. doi: 10.3389/fonc.2023.1326032 (PMC10715586; doi:10.3389/fonc.2023.1326032)

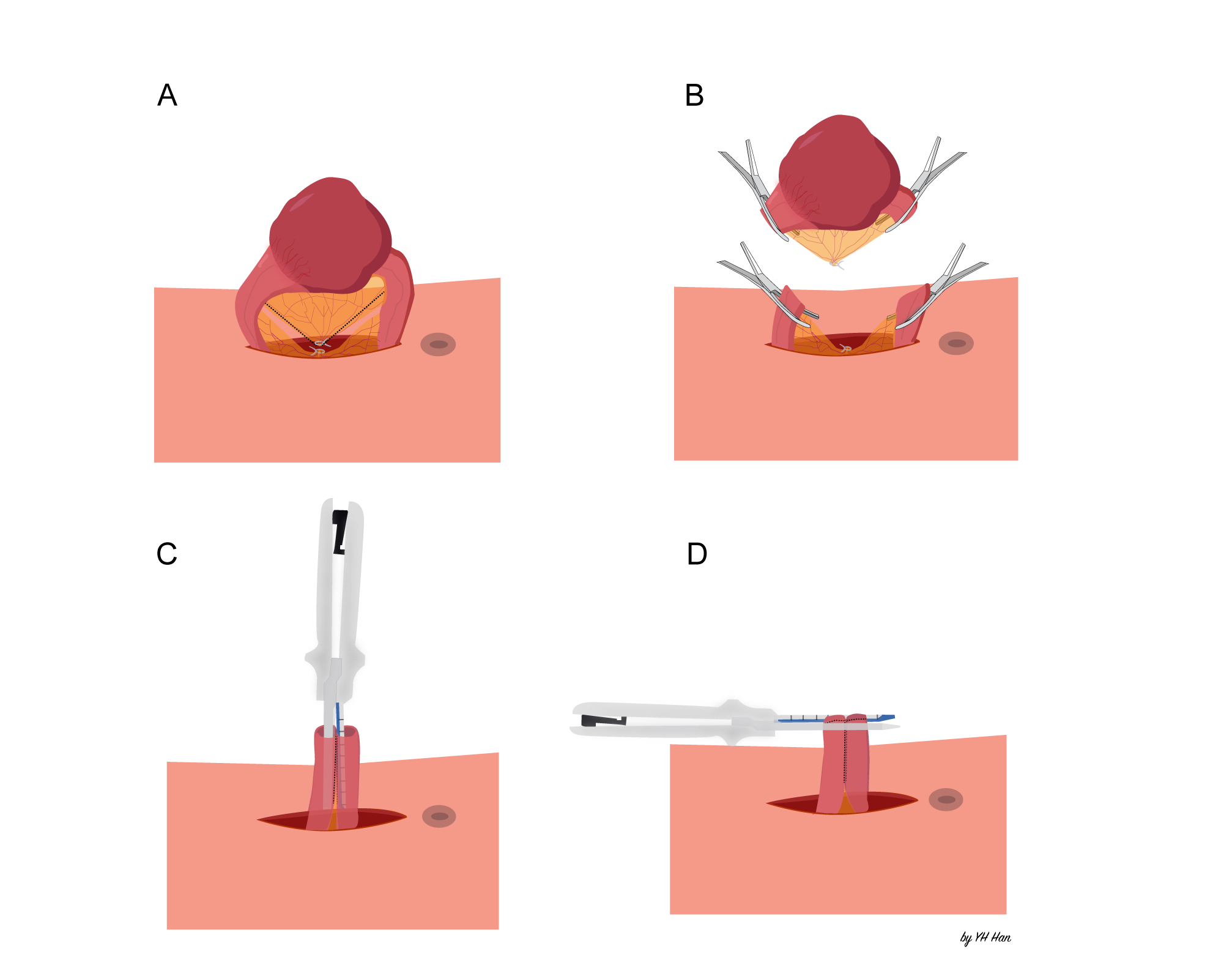

Supplement: Supplementary Figure 1 — The flow diagram of the surgical procedure, extracorporeal resection of the mass and segmental jejunum with primary side-to-side jejunojejunostomy. [file Image_1.tif]
